# Electromechanical Analysis of Flexoelectric Nanosensors Based on Nonlocal Elasticity Theory

**DOI:** 10.3390/mi11121077

**Published:** 2020-12-04

**Authors:** Yaxuan Su, Zhidong Zhou

**Affiliations:** 1 Chengyi University College, Jimei University, Xiamen 361021, China; suyaxuan@jmu.edu.cn; 2 Fujian Provincial Key Laboratory of Advanced Materials, College of Materials, Xiamen University, Xiamen 361005, China

**Keywords:** flexoelectricity, induced potential, nonlocal effect, sensors

## Abstract

Flexoelectric materials have played an increasingly vital role in nanoscale sensors, actuators, and energy harvesters due to their scaling effects. In this paper, the nonlocal effects on flexoelectric nanosensors are considered in order to investigate the coupling responses of beam structures. This nonlocal elasticity theory involves the nonlocal stress, which captures the effects of nonlocal and long-range interactions, as well as the strain gradient stress. Based on the electric Gibbs free energy, the governing equations and related boundary conditions are deduced via the generalized variational principle for flexoelectric nanobeams subjected to several typical external loads. The closed-form expressions of the deflection and induced electric potential (voltage) values of flexoelectric sensors are obtained. The numerical results show that the nonlocal effects have a considerable influence on the induced electric potential of flexoelectric sensors subjected to general transverse forces. Moreover, the induced electric potential values of flexoelectric sensors calculated by the nonlocal model may be smaller or larger than those calculated by the classical model, depending on the category of applied loads. The present research indicates that nonlocal effects should be considered in order to understand or design basic nano-electromechanical components subjected to various external loads.

## 1. Introduction

The nanoscale cantilever beam has been widely utilized as a basic element in nano-electromechanical systems, such as nanoactuators, nanosensors, and nano energy harvesters. At the nanoscale, the mechanical and electrical properties of nanostructures have significant differences from their bulk structures, which are size effects. In a few decades, flexoelectric, nonlocal, coupled stress, and strain gradient theories have been developed to capture the size effects for cases where the classical theories cannot describe the real electromechanical responses of the nanostructures.

Flexoelectricity, which is related to the electromechanical coupling between the polarization and the strain gradient, exists in a wide variety of dielectric materials and may lead to strong size-dependent properties at the nanoscale [1,2,3]. Recently, several experiments, theories, and applications related to the flexoelectric effect for dielectric materials, such as ferroelectric thin films, polymers, liquid crystals, and living membranes, have been published [4,5,6]. Based on previous work [7,8], Majdoub et al. [9] extended the static continuum theory to obtain the equations of motion and further discussed the electromechanical coupling responses of the nanobeams subjected to the strain gradient. Their results showed that the effective bending stiffness and effective piezoelectricity have a significant size effect due to flexoelectricity at the nanoscale. Furthermore, Shen and Hu [5] also established a more comprehensive theoretical framework using a variational principle for dielectrics by incorporating the flexoelectricity, electrostatic force, and surface effect. For flexoelectricity in membranes, Mohammadi et al. [10] developed a linear general framework of a homogenization problem, which can be used to analyze living membranes or liquid crystals. The effective elastic, dielectric, and flexoelectric properties of heterogeneous membranes were estimated in their paper. Employing these theories, the static and dynamic electromechanical responses of flexoelectric nanostructures and flexoelectric energy harvesters have been investigated [11,12,13,14,15,16,17,18]. Zhou et al. [15] investigated the electromechanical responses of flexoelectric beams with three different electrical boundary conditions, in which the induced electric potential was discussed in detail. We found that the maximum output electric potential is dependent on the aspect ratios of the beam and independent of the flexoelectric coefficients. Taking the strain gradient elastic effect into account, Lu et al. [19] gave an electromechanical coupling analysis of a bilayer flexoelectric nanobeam. In their study, the sharp gradient of the electric field existed near the beam surfaces due to the flexoelectric effect. Later, Su et al. [20] also investigated the influence of the piezoelectric effect on electromechanical coupling responses of a bilayer piezo-flexoelectric nanobeam due to the strain gradient elasticity. In that paper, the piezoelectric effect played a leading role in the induced large-scale electric potential, while the flexoelectric and strain gradient elastic effects dominated the induced electric potential at the nanoscale. Based on the general modified strain gradient theory, Chu et al. [21] established a mathematical framework to analyze bending and vibration responses of functionally graded piezo-flexoelectric nanobeams. The authors verified that the variation of the polarization density field along the thickness direction is fully dependent on the functionally graded model. Considering the flexoelectric effect, Xiang et al. [22] presented an exact elasticity solution for a functionally graded beam subjected to electromechanical loads. Recently, Wang et al. [6] published a new review of flexoelectricity in solids, in which they systematically discussed recent progress in both theoretical and experimental research in terms of flexoelectric effects.

In recent years, the nonlocal elastic field theory proposed by Eringen [23], which outlines the stress–strain relation at a point dependent on the strains of all points, has been considered for flexoelectric nanostructures. The nonlocal elasticity theory, which is common in nanostructures, can reasonably explicate the size effects of the static and dynamic behavior of nanobeams and nanoplates [24,25,26,27]. Taking the shear deformation into account, Li et al. [28] investigated the free vibration of the functionally graded Timoshenko beams with the nonlocal strain gradient effects. Their results showed that the vibration frequencies generally increase as the nonlocal parameters decrease. Based on the original elasticity model of nonlocal theory, without any additional assumptions, Li et al. [29] analyzed the bending of a nano-cantilever beam subjected to various mechanical loads. They found that whether the equivalent stiffness of the beam is weakened or strengthened depends on the competitive relation of the different external loads. Within the framework of the nonlocal elasticity theory, Ebrahimi and Barati [30] studied the vibration characteristics of the flexoelectric nanobeam considering the surface effect. Further, Barati [31] investigated the nonlinear vibration characteristics of flexoelectric nanobeams under the magnetic field due to the nonlocal and surface effects. In their paper, the closed-form nonlinear frequency of the flexoelectric nanobeam was obtained. Masoumi et al. [32] analyzed the flexoelectric effect on wave dispersion characteristics of piezoelectric nanobeams based on the nonlocal strain gradient theory. The static and dynamic mechanical behavior of flexoelectric nanobeams is significantly influenced by the nonlocal effect. The nonlocal effect causes the beam to be stiffer or softer, depending on the mechanical boundary conditions and category of applied mechanical loads. It is not clear how the coupling of flexoelectric and nonlocal effects acts on the electromechanical responses of sensors. However, to our knowledge, none of the previous papers on flexoelectric sensors covering surface electrodes has considered the nonlocal effect. Therefore, it is beneficial to analyze the induced electric potential of flexoelectric sensors, which are subjected to various mechanical loads, incorporating the nonlocal effect.

In this paper, the objective is to deal with the nonlocal effect on the induced electric potential of flexoelectric sensors subjected to various mechanical loads. Introducing the nonlocal elasticity theory into the linear piezo-flexoelectric model, the equilibrium equations of nanosensors and the corresponding general mechanical boundary conditions are derived using the generalized variational method. The influences of structural sizes, nonlocal parameters, and types of external loads on the normalized deflection and induced electric potential are presented graphically and discussed.

## 2. Nonlocal Elasticity Theory of Flexoelectric Materials

Taking the nonlocal elasticity theory into account, the linear piezo-flexoelectric theory is employed to analyze the electromechanical coupling responses of flexoelectric nanostructures. In the nonlocal elasticity model, which contains a wide range interaction, the stress at a point is a function of the strains of all neighboring points [28,33]. Hence, the stress and electric displacement tensors can be expressed as [28,30,31,33]:

(1) $$\left(1-{\left(e_{0}a\right)}^{2}\nabla^{2}\right)\sigma_{ij}=c_{ijkl}\varepsilon_{kl}-e_{kij}E_{k}$$

(2) $$\left(1-{\left(e_{0}a\right)}^{2}\nabla^{2}\right)\sigma_{ijk}=-\mu_{lijk}E_{l}+g_{ijklmn}\varepsilon_{lm,n}$$

(3) $$D_{i}=\kappa_{ij}E_{j}+e_{ijk}\varepsilon_{jk}+\mu_{ijkl}\varepsilon_{jk,l}$$

where $\sigma_{ij}$ and $\sigma_{ijk}$ are the normal stress tensor and higher-order stress tensor of nonlocal theory and $D_{i}\;$is the electric displacement vector; $e_{0}a$ is the nonlocal parameter, which is dependent on the special material and internal characteristic scale; $c_{ijkl}$ and $\kappa_{ij}$ are the fourth-rank elastic modulus and the second-rank dielectric constant, respectively; $e_{ijk}$ is the third-rank piezoelectric tensor; $\mu_{ijkl}$ is the fourth-rank flexoelectric tensor; $g_{ijklmn}$ is the sixth-rank strain gradient elastic tensor, which is dependent on the strain gradient elastic theory; $\varepsilon_{ij}$ is the strain tensor, $E_{i}$ is the electric field vector, and $\varepsilon_{jk,l}$ is the strain gradient tensor; ∇ is the Laplacian operator. Here, the Kleinert and Gauge theory [34] is used. For isotropic materials, the expression of $g_{ijklmn}$ is [19,20]:

(4) $$\begin{array}{c} g_{ijklmn}=c_{1111}l_{1}^{2}\delta_{ij}\delta_{kn}\delta_{lm}+c_{1212}l_{2}^{2}(4\delta_{il}\delta_{jk}\delta_{mn}\delta_{kn}-4\delta_{in}\delta_{jk}\delta_{mn}\delta_{kl}\\ +\delta_{ij}\delta_{kn}\delta_{lm}\delta_{il}-\delta_{ij}\delta_{kn}\delta_{lm}) \end{array}$$

where $\delta_{ij}=1\left(i=j\right),\;\delta_{ij}=0\left(i\neq j\right)$ is the Dirac delta function and $l_{1},l_{2}$ are two material scale parameters with length dimensions.

The total electrical enthalpy of flexoelectric solids can then be written as [14,15]:

(5) $$\Pi =∭\;Gdv-∯t_{i}u_{i}ds-∯r_{i}v_{i}ds+∯\varpi \phi ds$$

where G is the Gibbs free energy density; $t_{i}$ and $u_{i}$ are the traction and displacement on the surface, respectively; $r_{i}$ and $v_{i}$ are the higher-order traction and normal derivative of displacement on the surface, respectively; ϖ and ϕ are the surface charge density and electric potential, respectively. In general, $r_{i}=\sigma_{ijk}n_{j}n_{k}$ and $v_{i}=u_{i,j}n_{j}$, where $n_{j}$ is the outward unit normal vector on the surface [5,15].

## 3. Theoretical Formulation of Flexoelectric Sensors with the Nonlocal Effect

**Figure 1:**
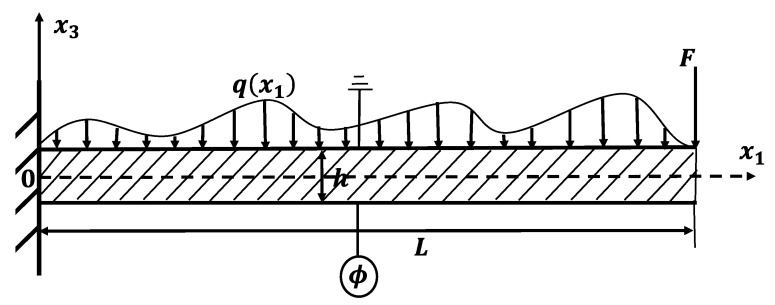
A cantilever beam model of the flexoelectric sensor subjected to external loads.

In this paper, a cantilever beam, in which h, b, and *L* are the thickness, width, and length, respectively, is depicted in Figure 1. The beam is mechanically fixed at the left end and loaded by distributed lateral forces $q\left(x_{1}\right)$, with a concentrated force at the right end. In a Cartesian coordination system, the *x*_1_-axis is coincident with the neutral surface and the *x*_3_-axis runs along the thickness direction of the beam. The bottom surface electrode undergoes a change of electric potential as a result of mechanical deformation and the top electrode is grounded.

As the beam is slender (h≪L), the Bernoulli–Euler beam assumption is used to model the flexoelectric sensor. The strain and strain gradient fields at any point can be taken as follows [14,15,20]:

(6) $$\varepsilon_{11}=-x_{3}\frac{d^{2}w}{dx_{1}^{2}},\;\;\varepsilon_{11,3}=-\frac{d^{2}w}{dx_{1}^{2}},\;\;\;\;\;\varepsilon_{11,1}=-x_{3}\frac{d^{3}w}{dx_{1}^{3}}$$

where w is the displacement in the $x_{3}$ direction, while the displacement in the $x_{2}$ direction is set to zero, as in plane strain elasticity. In the present Bernoulli–Euler beam model, the strain gradient $\varepsilon_{11,1}$ is very small compared to the strain gradient $\varepsilon_{11,3}$ and may be neglected for a slender beam. The electric field in a slender beam is predominant in the thickness direction, while the electric field component in the length direction ($E_{1}$) is negligible [14,15,30,31]. For the slender beam, the general constitutive equations (Equations (1)–(3)) can be further simplifies as:

(7) $$\{\begin{array}{c} \left[1-{\left(e_{0}a\right)}^{2}\frac{d^{2}}{dx_{1}^{2}}\right]\sigma_{11}=c_{11}\varepsilon_{11}-e_{311}E_{3}\;\;\;\;\;\;\;\\ \left[1-{\left(e_{0}a\right)}^{2}\frac{d^{2}}{dx_{1}^{2}}\right]\sigma_{113}=-\mu_{13}E_{3}+g_{13}\varepsilon_{11,3}\\ D_{3}=\kappa_{33}E_{3}+e_{311}\varepsilon_{11}+\mu_{13}\varepsilon_{11,3}\;\;\;\;\;\;\;\;\;\;\;\;\;\;\;\;\;\; \end{array}$$

where the subscripts of the material property tensors are contracted for simplicity [19], i.e., $c_{11}=c_{1111}$,$\;\mu_{13}=\mu_{3113}$,$\;g_{13}=g_{113113}$. The electric potential $\Phi \left(x_{1},x_{3}\right)$ in the beams shown in Figure 1 is related to the electric field by $E_{3}=-\partial \Phi /\partial x_{3}$. In the absence of free body charges, Gauss’s law of electrostatics requires $D_{3,3}=0$. In Figure 1, $\Phi \left(x_{3}=h/2\right)=0$ and $\Phi \left(x_{3}=-h/2\right)=\phi \left(x_{1}\right)$ are on the top and bottom surfaces of the flexoelectric beam, respectively; $\phi \left(x_{1}\right)$ is the induced electric potential due to the flexoelectric effect. Solving Gauss’s equation, the electric field can be expressed as [15,20]:

(8) $$E_{3}=-\frac{e_{311}}{\kappa_{33}}\varepsilon_{11}+\frac{\phi \left(x_{1}\right)\;}{h}$$

For the flexoelectric beam with the nonlocal effect, the virtual general electric Gibbs free energy δG can be expressed as [14,28,33]:

(9) $$\begin{array}{c} \delta G=\int_{V}\left(\sigma_{11}\delta \varepsilon_{11}+\sigma_{113}\delta \varepsilon_{11,3}-D_{3}\delta E_{3}\right)dV\\ =-\int_{V}\left[\sigma_{11}\delta \left(x_{3}\frac{d^{2}w}{dx_{1}^{2}}\right)+\sigma_{113}\delta \left(\frac{d^{2}w}{dx_{1}^{2}}\right)+D_{3}\delta \left(\frac{e_{311}}{\kappa_{33}}x_{3}\frac{d^{2}w}{dx_{1}^{2}}+\frac{\phi \left(x_{1}\right)\;}{h}\right)\right]dV \end{array}$$

where V is volume of the beam. The following stress resultant and electric displacement resultant are considered:

(10) $$M=\int_{A}x_{3}\sigma_{11}dA,\;P=\int_{A}\sigma_{113}dA,\;D=\int_{A}D_{3}dA,\;\bar{D}=\int_{A}x_{3}D_{3}dA$$

where A=bh is the area of the cross-section. Substituting Equations (6) and (8) into the third item of Equation (7), the electric displacement can be rewritten as:

(11) $$D_{3}=\frac{\kappa_{33}}{h}\phi \left(x_{1}\right)-\mu_{13}\frac{d^{2}w}{dx_{1}^{2}}$$

The above electric displacement is constant along the thickness direction of the beam, so $\bar{D}=0$. The virtual work of external electromechanical loads δW can be expressed as:

(12) $$\delta W=\int_{L}\left[q\left(x_{1}\right)\delta w-b\varpi \delta \phi \right]dL+F\delta w$$

According to the generalized variational principle, we get δΠ=0 for the mechanical and electrostatic equilibrium of the flexoelectric actuators and sensors due to the arbitrariness of δw and δϕ:

(13) $$\delta w:\;\frac{d^{2}M}{dx_{1}^{2}}+\frac{d^{2}P}{dx_{1}^{2}}+q\left(x_{1}\right)=0$$

(14) δϕ: D−ϖb=0

The corresponding electromechanical coupling boundary conditions can be given by:

(15) $$\{\begin{array}{c} w\;or\;\frac{dM}{dx_{1}}+\frac{dP}{dx_{1}}\\ \frac{dw}{dx_{1}}\;or\;M+P \end{array}$$

Integrating both sides of the first and second items of Equation (7) along the thickness of the beam, the constitutive equation can be rewritten as:

(16) $$\{\begin{array}{c} \left[1-{\left(e_{0}a\right)}^{2}\frac{d^{2}}{dx_{1}^{2}}\right]M=-G_{P}\frac{d^{2}w}{dx_{1}^{2}}\;\\ \left[1-{\left(e_{0}a\right)}^{2}\frac{d^{2}}{dx_{1}^{2}}\right]P=-\mu_{13}b\phi \left(x_{1}\right)-g_{13}A\frac{d^{2}w}{dx_{1}^{2}} \end{array}$$

where $G_{P}=\frac{bh^{3}}{12}\left(c_{11}+\frac{e_{311}^{2}}{\kappa_{33}}\right)$ is the effective bending rigidity of the piezoelectric nanobeam without considering the flexoelectricity [15]. Applying Equations (13) and (16), the stress resultant can be obtained:

(17) $$M+P=-G_{S}\frac{d^{2}w}{dx_{1}^{2}}-\mu_{13}b\phi \left(x_{1}\right)-{\left(e_{0}a\right)}^{2}q\left(x_{1}\right)$$

where $G_{S}=G_{P}+g_{13}A$ is the effective bending rigidity of the piezoelectric beam with the strain gradient elasticity [20]. Using Equation (17), we obtain:

(18) $$\frac{d^{2}}{dx_{1}^{2}}\left(M+P\right)=-G_{S}\frac{d^{4}w}{dx_{1}^{4}}-\mu_{13}b\frac{d^{2}\phi \left(x_{1}\right)}{dx_{1}^{2}}-{\left(e_{0}a\right)}^{2}\frac{d^{2}q\left(x_{1}\right)}{dx_{1}^{2}}$$

Substituting Equation (18) into Equation (13), the mechanical equilibrium of the flexoelectric beam with the nonlocal effect can be rewritten as:

(19) $$G_{S}\frac{d^{4}w}{dx_{1}^{4}}+\mu_{13}b\frac{d^{2}\phi \left(x_{1}\right)}{dx_{1}^{2}}+{\left(e_{0}a\right)}^{2}\frac{d^{2}q\left(x_{1}\right)}{dx_{1}^{2}}-q\left(x_{1}\right)=0$$

Equation (19) can describe the electromechanical responses of flexoelectric beams with different electrical boundary conditions. We have discussed these electrical boundary conditions in detail [15]. The Open Circuit(OC) condition: A beam without surface electrodes operates under an open circuit, in which the surface electric potential is dependent on $x_{1}$. The Closed Circuit with Fixed voltage(CCF) condition: A beam with surface electrodes is subjected to an external voltage, such as an actuator. The Open Circuit with Induced electric potential(OCI) condition: A beam with surface electrodes operates under an open circuit, in which the surface electric potential is induced by the mechanical deformation, such as a sensor or an energy harvester.

In the present case, the flexoelectric sensors would be considered. We note that the top and bottom surfaces of the flexoelectric beam are covered with electrodes perfectly, meaning the electric potential $\phi \left(x_{1}\right)$ is independent on $x_{1}$. For flexoelectric sensors or actuators, since δϕ is independent of $x_{1}$ in Equation (14), we obtain $\int_{L}\left(D-\varpi b\right)dA=0$ [15]. Moreover, $\int_{0}^{L}\varpi bdx_{1}=0$ should be satisfied (no supply of charges to the electrodes) on the surfaces of sensors. Therefore, the induced electric potential can be obtained from Equation (11) as:

(20) $$\phi =\frac{\mu_{13}h}{\kappa_{33}L}\frac{dw}{dx_{1}}{|}_{0}^{L}$$

The induced electric potential as a result of mechanical deformation due to the flexoelectric effect is proportional to the deviation between two rotational angles at the ends. Applying Equations (15), (17), (19), and (20), the nonlocal equilibrium equation and the corresponding general mechanical boundary conditions for the cantilever beams can be expressed as:

(21) $$\left\{ \begin{array}{l} 0<x_{1}<L:\;G_{S}\frac{d^{4}w}{dx_{1}^{4}}+{\left(e_{0}a\right)}^{2}\frac{d^{2}q\left(x_{1}\right)}{dx_{1}^{2}}-q\left(x_{1}\right)=0\\ x_{1}=0:\;w=\frac{dw}{dx_{1}}=0\\ x_{1}=L:\;G_{S}\frac{d^{3}w}{dx_{1}^{3}}+{\left(e_{0}a\right)}^{2}\frac{dq\left(x_{1}\right)}{dx_{1}}=-F;G_{S}\frac{d^{2}w}{dx_{1}^{2}}+\frac{\mu_{13}^{2}A}{\kappa_{33}L}\frac{dw}{dx_{1}}+{\left(e_{0}a\right)}^{2}q\left(x_{1}\right)=0 \end{array}\right.$$

Equation (21) can be easily simplified into equations of the nonlocal strain gradient theory for elastic materials [28,29] by setting $\mu_{13}=\kappa_{33}=e_{311}=0$ in $G_{S}$, and can also be obtained using the classical piezo-flexoelectric bending equations by setting the nonlocal parameter $e_{0}a$ as zero.

## 4. Numerical Results and Discussion

In order to assess nonlocal and flexoelectric effects, we discuss bending beams subjected to distributed loads and a concentrated force. In this simulation, the material is BaTiO_3_ and the corresponding material parameters are $c_{11}=131\;\mathrm{GPa}$, $\kappa_{33}=12.56\;\mathrm{nC}/\left(V\cdot m\right)$, $e_{311}=-4.4\;C/m^{2}$, $\mu_{13}=$10^−6^ C/m, and $g_{13}=c_{11}l^{2}$. Here, the internal material length scale parameter l=1.92 nm [19]. The slenderness ratio of the cantilever flexoelectric sensor is fixed to *L/h* = 20 and the width *b = h*, unless otherwise stated.

### 4.1. Subjected to Uniformly Distributed Loads and a Concentrated Force

Solving the general nonlocal equilibrium and boundary conditions (Equation (21)), the deflection w of the cantilever beam with uniformly distributed loads $q_{0}$ and a concentrated force F can be obtained as:

(22) $$\begin{array}{c} w=\frac{q_{0}}{G_{S}}\left(\frac{x_{1}^{4}}{24}-\frac{x_{1}^{3}L}{6}+\frac{x_{1}^{2}L^{2}}{4}\right)-\frac{F}{6G_{S}}\left(x_{1}^{3}-3x_{1}^{2}L\right)-\frac{\alpha +6\tau^{2}}{12G_{S}\left(1+\alpha \right)}q_{0}x_{1}^{2}L^{2}\\ -\frac{\alpha }{4G_{S}\left(1+\alpha \right)}Fx_{1}^{2}L \end{array}$$

where $\tau =e_{0}a/L$ is the dimensionless scaling effect factor accounting for the nonlocal effect [26,27,28] and $\alpha =\left(\mu_{13}^{2}A\right)/\left(\kappa_{33}G_{S}\right)$. It can be seen from Equation (22) that the bending beam with nonlocal and flexoelectric effects undergoes a smaller deflection than a beam without these effects subjected to the same loads. Substituting Equation (22) into Equation (20), the induced electric potential of cantilever beams with $q_{0}$ and F can be expressed as:

(23) $$\phi =\frac{\mu_{13}hL}{2\kappa_{33}}\frac{1}{G_{S}\left(1+\alpha \right)}\left[\frac{1-6\tau^{2}}{3}q_{0}L+F\right]$$

Equation (23) shows that the induced electric potential is entirely dependent on the nonlocal effect when only uniformly distributed loads exist. No nonlocal effect arises for the induced electric potential of flexoelectric sensors subjected to a concentrated force only.

**Figure 2:**
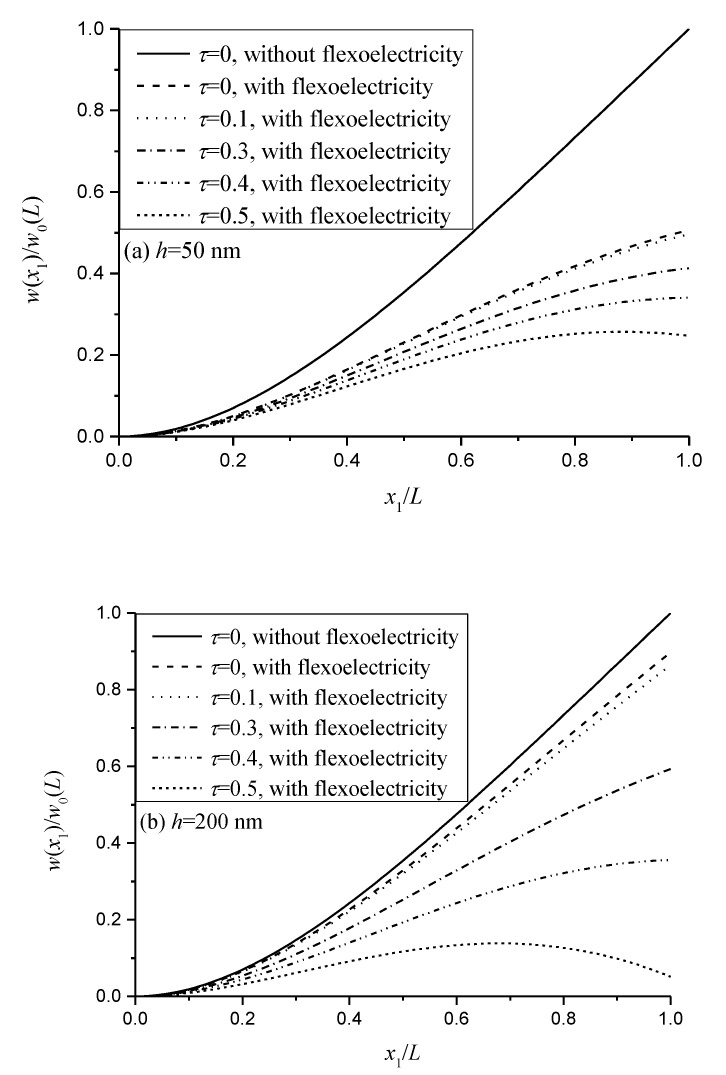
Nonlocal and flexoelectric effects on the normalized deflection of the nanobeam for different thicknesses: (**a**) *h =* 50 nm; (**b**) *h =* 200 nm.

In Figure 2, the normalized deflection $w\left(x_{1}\right)/w_{0}\left(L\right)$ for the cantilever beam is illustrated by the different nonlocal parameters subjected to $q_{0}$ only, in which $w_{0}\left(L\right)=q_{0}L^{4}/8G_{S}$ is the deflection at the free end without the nonlocal and flexoelectric effects. The flexoelectric and nonlocal effects cause the sensor to stiffen. By increasing the nonlocal parameter τ, the deflection decreases, which means that the nonlocal effect causes the beam to stiffen with the uniformly distributed loads. This result could also be found from Equation (22). Comparing the results in Figure 2a,b, it can be seen that the bending beam has a larger stiffness for the larger thickness with a large nonlocal parameter τ. Obviously, the flexoelectric effect could increase the effective stiffness of the flexoelectric sensors shown in Figure 2. The results from Majdoub et al. [9] also showed that the normalized Young’s modulus of lead zirconate titanate (PZT) beams increases as the thickness of the beams decreases due to the flexoelectric effect. The normalized deflection of PZT-5H beams with the flexoelectric effect is also smaller than that without the flexoelectric effect [14]. Abdollahi et al. [35] found the enhanced size-dependent elasticity due to the flexoelectric effect by applying the smooth mesh-free basis functions in a Galerkin method. In their paper, the normalized stiffness of the flexoelectric cantilever beams of BaTiO_3_ under the OCI condition increased as the normalized thickness decreased.

**Figure 3:**
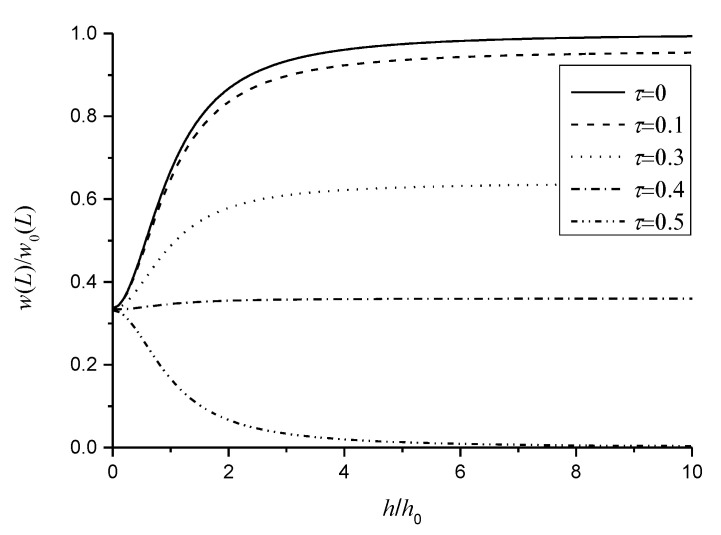
Variation of the normalized deflection at the free end with beam-normalized thickness for different nonlocal parameters.

The boundary condition in Equation (21) indicates that nonlocal and flexoelectric effects act as a concentrated bending moment at the free end, which would change the shape of the deflection curve, especially near the free end. Hence, Figure 3 is presented to investigate the normalized deflection $w\left(L\right)/w_{0}\left(L\right)$ at the free end of sensors versus the normalized thickness $h/h_{0}$ for the different nonlocal parameters, in which $h_{0}=\sqrt{12\mu_{13}^{2}/\left(c_{11}\kappa_{33}+e_{311}^{2}\right)}\;$denotes an intrinsic thickness for the maximum induced electric potential of flexoelectric beams [15]. When τ=0, the result is identical to the results of flexoelectric sensors [20]. It can also be observed that the normalized deflections at the free end decrease as the nonlocal parameter increases, then decrease rapidly to the same value (as h→0) with the decrease of the beam-normalized thickness for τ=0.1, 0.3. When τ=0.4, the variation of the normalized deflection is almost independent of $h/h_{0}$. However, when τ=0.5, the normalized deflection at the free end increases from zero to approach the same value (as h→0) as the normalized thickness decreases.

**Figure 4:**
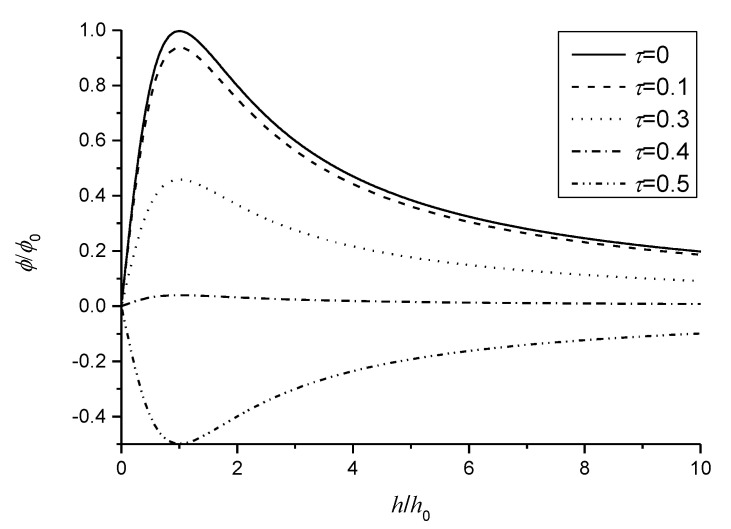
Variation of the normalized induced electric potential with the normalized thickness for different nonlocal parameters.

The induced electric potential, which is very important for sensors and energy harvesters, has a significant scaling effect. A maximum value exists for the intrinsic thickness $h_{0}$ in flexoelectric sensors without the nonlocal effect [15,20]. Figure 4 plots the normalized induced electric potential $\phi /\phi_{0}$ ($\phi_{0}=q_{0}L^{2}/\left[A\sqrt{12\left(c_{11}\kappa_{33}+e_{311}^{2}\right)}\right]$ is the maximum amplitude of the induced electric potential of flexoelectric sensors [15]) of the nonlocal model as a function of the normalized sensor thickness subjected to $q_{0}$ only. Kwon et al. [36] theoretically verified that the normalized charge output of the flexo-piezoelectric multilayered structure of barium strontium titanate (BST) increases as the thickness decreases under force input conditions. The induced electric potential due to the nonlocal effect decreases because the rotational angles at the free end decrease, as shown in Figure 2. It is observable from Equation (23) that when $\tau^{2}>1/6$, the induced electric potential due to the flexoelectric effect changes the direction in the sensors, as shown in Figure 4. It is interesting that no nonlocal effect arises for the intrinsic thickness $h_{0}$, in which there exists a maximum induced electric potential.

**Figure 5:**
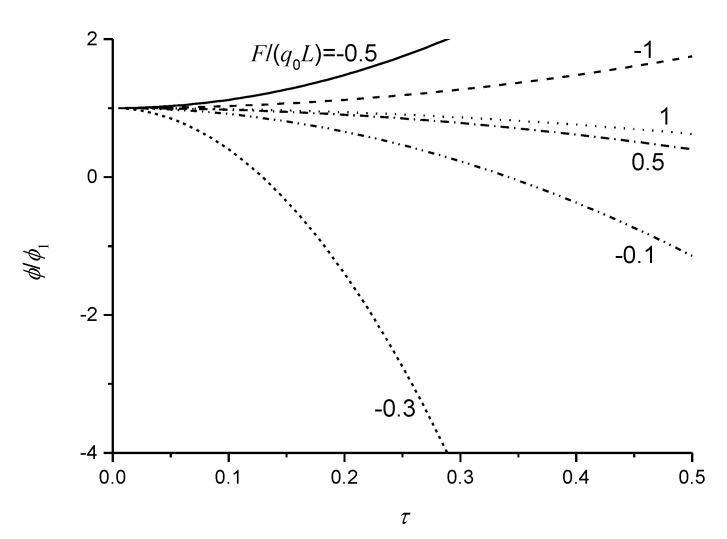
Variation of the normalized induced electric potential with the nonlocal parameter for different combinations of uniformly distributed loads and a concentrated force.

From Equation (23), it can be clearly found that neglecting the nonlocal effect causes the induced electric potential to disappear as $F/(q_{0}L)=-1/3$. Figure 5 illustrates the normalized induced electric potential $\phi /\phi_{1}$, where $\phi_{1}$ is the induced electric potential of flexoelectric sensors subjected to $q_{0}$ and *F* without the nonlocal effect, as a function of the nonlocal parameter. It is observable from Figure 5 that the combination of $q_{0}L$ and *F* have a great influence on the induced electric potential of the flexoelectric sensors due to the nonlocal effect. When $F/(q_{0}L)>-1/3$, the normalized induced electric potential decreases and could change the sign by increasing the nonlocal parameter. However, when $F/(q_{0}L)<-1/3$, the normalized induced electric potential would increase with the increase of the nonlocal parameter. For given materials with the nonlocal effect, the category $F/(q_{0}L)<-1/3$ should be chosen to obtain better electric responses.

### 4.2. Subjected to Sinusoidal Distributed Loads and a Concentrated Force

When the uniformly distributed load $q_{0}$ has been considered, the expression of $d^{2}q\left(x_{1}\right)/dx_{1}^{2}=0$ should be satisfied in Equation (21). Here, a concentrated force F at the free end and transverse distributed loads $q=q_{0}\sin\left(n\pi x_{1}/L\right)$ are applied on the nano-cantilever beams, where n=1, 2, 3,…. Applying Equation (21) with F and q, the bending equation can be given by:

(24) $$\frac{d^{4}w}{dx_{1}^{4}}-\lambda \sin\left(\frac{n\pi x_{1}}{L}\right)=0$$

where $\lambda =q_{0}\left(\pi^{2}n^{2}\tau^{2}+1\right)/G_{S}$. By solving Equation (24) with the general mechanical boundary conditions in Equation (21), the expression of the deflection is obtained as:

(25) $$w=\lambda {\left(\frac{L}{n\pi }\right)}^{4}\sin\left(n\pi x_{1}/L\right)+\frac{C_{3}}{6}x_{1}^{3}+\frac{C_{2}}{2}x_{1}^{2}+C_{1}x_{1}$$

where:

(26) $$C_{1}=-\lambda {\left(\frac{L}{n\pi }\right)}^{3},\;\;C_{3}=\frac{\left[\frac{q_{0}L}{n\pi }{\left(-1\right)}^{n}-F\right]}{G_{S}},\;C_{2}=\frac{-C_{3}L-\alpha \left[\frac{C_{3}}{2}L^{2}+C_{1}\left(1-{\left(-1\right)}^{n}\right)\right]}{1+\alpha }$$

The rotational angle at the free end can be written as:

(27) $$\frac{dw}{dx_{1}}{|}_{L}=C_{1}\left(1-{\left(-1\right)}^{n}\right)+\frac{C_{3}}{2}L^{2}+C_{2}L$$

**Figure 6:**
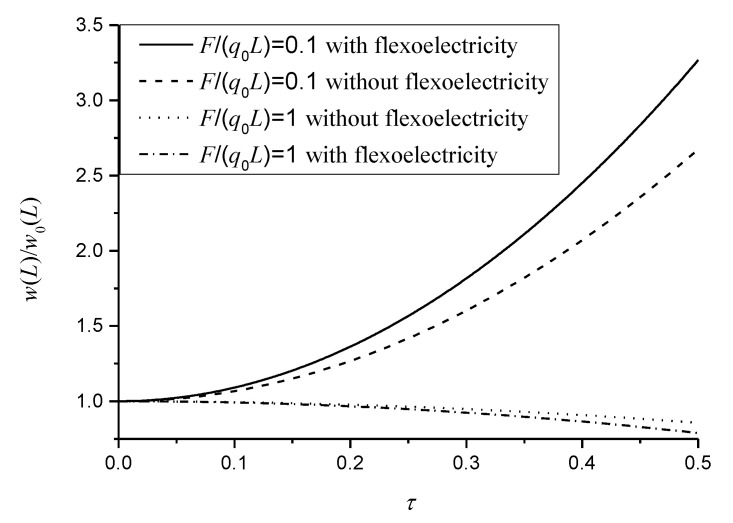
Variation of the normalized deflection at the free end subjected to a combination of sinusoidal distributed loads and a concentrated force.

When n is an even number, the rotational angle expressed in Equation (27) is independent of the nonlocal parameter τ. This means that the nonlocal effect has no influence on the induced electric potential as n=2, 4, 6,…. Figure 6 plots the relationship between the normalized deflection and the nonlocal parameter with and without the flexoelectric effect, in which n=2. In Figure 6, we can observe that the ratio of the concentrated force and distributed loads determines the normalized deflection as being higher or lower than that of the classical model. At these two kinds of loads, the bending deformations of cantilever beams without the flexoelectric effect have different directions, as described in Equation (30) of the literature [29]. However, the effective mechanical moment induced by the converse flexoelectric effect has the same direction in the bending deformation of the flexoelectric sensors [15,20,35]. Hence, the flexoelectric effect would further strengthen ($F/(q_{0}L)=1$) or weaken ($F/(q_{0}L)=0.1$), as in the bending stiffness of flexoelectric sensors involving the nonlocal effect.

**Figure 7:**
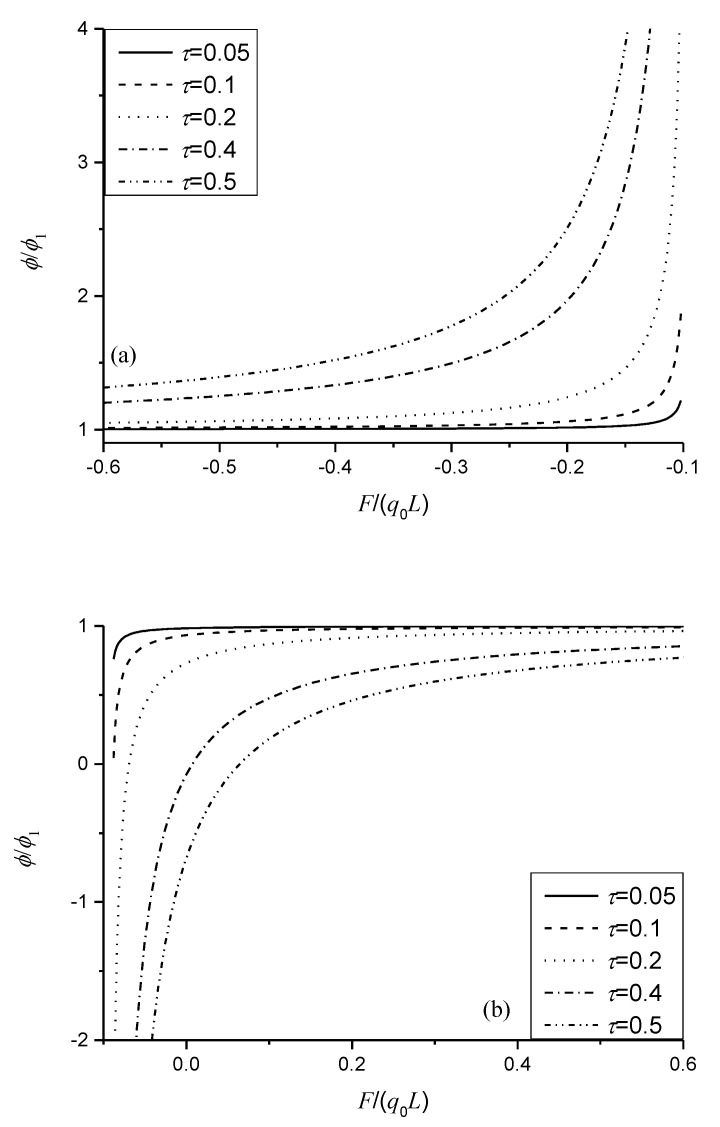
Variation of the normalized induced electric potential with $F/(q_{0}L)$ for different nonlocal parameters: (**a**) $F/(q_{0}L)<-0.1$; (**b**) $F/(q_{0}L)>-0.1$.

By taking n=1, the nonlocal effect has a significant influence on the induced electric potential shown in Equation (27). Figure 7 is presented to investigate the nonlocal effect on variation of the normalized induced electric potential with respect to $F/(q_{0}L)$. When $F/(q_{0}L)<-0.1$, the induced electric potential is always strengthened and increases with the increase of the nonlocal parameter and the ratio of $F/(q_{0}L)$, shown in Figure 7a. From Figure 7b, we can also observe that the induced electric potential is weakened by some ratios of $F/(q_{0}L)$ and decreases with the increase of the nonlocal parameter with some ratios of $F/(q_{0}L)$. However, the induced electric potential with the nonlocal effect would change the direction along the thickness of the sensors when the ratio of $F/(q_{0}L)$ approaches –0.1. This result indicates that the sign of the induced electric potential is fully dependent on the nonlocal effect and the category and nature of the external loads, which would be helpful in understanding or designing nanosensors in which the beam structures act as the basic elements. For given loads such as $F/(q_{0}L)<-0.1$, materials with large nonlocal parameters could be used to design better sensors. As $F/(q_{0}L)>-0.1$, materials with large nonlocal parameters can be used only when $F/(q_{0}L)$ approaches −0.1. Otherwise, materials with small nonlocal parameters are better options.

## 5. Conclusions

Flexoelectric sensors were investigated in the present paper considering the nonlocal effect. The governing equations and corresponding boundary conditions were developed based on the generalized variational principle of the flexoelectric nanobeams. Further, the expression of the induced electric potential, which is fully dependent on the rotational angles at the ends, was obtained. The numerical examples revealed that the induced electric potential of the flexoelectric sensors has a significant nonlocal effect with distributed loads only or with a combination of distributed loads and a concentrated force. Specifically, the nonlocal effect affects the induced electric potential of flexoelectric sensors with some combinations of external mechanical loads. Hence, the optimal induced electric potential of flexoelectric sensors can be obtained by choosing appropriate structural sizes, material parameters, and external mechanical loads. The results show that the electromechanical responses of flexoelectric sensors are sensitive to the nonlocal effect, which cannot be ignored. The present research could, thus, inspire theoretical and experimental work on new flexoelectric structures at the nanoscale.
